# Predicting postural control adaptation measuring EEG, EMG, and center of pressure changes: BioVRSea paradigm

**DOI:** 10.3389/fnhum.2022.1038976

**Published:** 2022-12-15

**Authors:** Simon A. Stehle, Romain Aubonnet, Mahmoud Hassan, Marco Recenti, Deborah Jacob, Hannes Petersen, Paolo Gargiulo

**Affiliations:** ^1^Institute of Biomedical and Neural Engineering, Reykjavik University, Reykjavik, Iceland; ^2^MINDig, Rennes, France; ^3^Department of Anatomy, Faculty of Medicine, School of Health Sciences, University of Iceland, Reykjavik, Iceland; ^4^Akureyri Hospital, Akureyri, Iceland; ^5^Department of Science, Landspitali, National University Hospital of Iceland, Reykjavik, Iceland

**Keywords:** postural control, machine learning, virtual reality, EEG, EMG, center of pressure

## Abstract

**Introduction:** Postural control is a sensorimotor mechanism that can reveal neurophysiological disorder. The present work studies the quantitative response to a complex postural control task.

**Methods:** We measure electroencephalography (EEG), electromyography (EMG), and center of pressure (CoP) signals during a virtual reality (VR) experience called BioVRSea with the aim of classifying different postural control responses. The BioVRSea paradigm is based on six different phases where motion and visual stimulation are modulated throughout the experiment, inducing subjects to a different adaptive postural control strategy. The goal of the study is to assess the predictability of those responses. During the experiment, brain activity was recorded from a 64-channel EEG, muscle activity was determined with six wireless EMG sensors placed on lower leg muscles, and individual movement measured by the CoP. One-hundred and seventy-two healthy individuals underwent the BioVRSea paradigm and 318 features were extracted from each phase of the experiment. Machine learning techniques were employed to: (1) classify the phases of the experiment; (2) assess the most notable features; and (3) identify a quantitative pattern for healthy responses.

**Results:** The results show that the EEG features are not sufficient to predict the distinct phases of the experiment, but they can distinguish visual and motion onset stimulation. EMG features and CoP features, when used jointly, can predict five out of six phases with a mean accuracy of 74.4% (±8%) and an AUC of 0.92. The most important feature to identify the different adaptive strategies is the Squared Root Mean Distance of points on Medio-Lateral axis (RDIST_ML).

**Discussion:** This work shows the importance and the feasibility of a quantitative evaluation in a complex postural control task and demonstrates the potential of EEG, CoP, and EMG for assessing pathological conditions. These predictive systems pave the way for developing an objective assessment of pathological behavior PC responses. This will be a first step in identifying individual disorders and treatment options.

## Introduction

An upright posture is not only important when standing or walking, it is also crucial for successfully accomplishing everyday life tasks and therefore has a major impact on the quality of life. Falls are the worst consequences of postural control (PC) disorders (Alexander, 1994), and for this reason, fall injuries are one of the most serious healthcare problems and one of the biggest threats to the independence of older adults. They are associated with an increased functional impairment, disability, and decreased ability to independently manage activities of daily living (Hageman et al., 1995; Marks et al., 2003; Figueiro et al., 2008).

PC can be defined as the ability to maintain the body’s center of gravity within certain limits of stability during quiet stance or movement. The limits of stability are shaped like a cone and can be specified as the range in which the body’s center of gravity can be shifted without requiring a change in the base of support (Alexander, 1994; Hageman et al., 1995, p. 961; Horak, 2006). PC involves two general skills of the human body: first, postural orientation, which describes the ability to maintain the body position in relation to the environment and ultimately provide an appropriate response to external perturbations; second, postural stability, which is the ability to maintain body position in equilibrium (Hageman et al., 1995, p. 961; Horak, 2006, 2; Figueiro et al., 2008, p. 111).

PC is a complex and dynamic central sensorimotor system that integrates information from the vestibular, visual, and proprioceptive sensory systems. In daily activities, it relies on a multifaceted interplay of physiological mechanisms (Melzer et al., 2001, p. 189; Peterka, 2002, p. 1,097; Horak, 2006; Ivanenko and Gurfinkel, 2018, p. 175).

External perturbations induce and trigger different adaptive PC strategies in the human body. Previous studies investigated these strategies, focusing on the task-dependent changes of strategies throughout the lifespan (Haddad et al., 2013), the adaptive behavior of PC due to perturbations in the upright standing (He and Tian, 1997; Fransson et al., 2000), or the adaptive responses to virtual environments (Reed-Jones et al., 2008).

A person’s CoP and its movement are indicators of stability and are considered particularly useful to study postural response. The CoP can be calculated using a force plate under the feet that determines the center of the vertical reaction force (Alexander, 1994; Hageman et al., 1995, p. 961). In addition, an increasing number of studies are using brain electrical signals (EEG) as a viable measurement setup to investigate cortical activity and the neurophysiological behavior during PC tasks (Slobounov et al., 2005, p. 316; Edwards et al., 2018, p. 36). Likewise, EMG has been used in several studies to investigate balance control as well as posture correction by lower leg muscles like tibialis anterior and gastrocnemius (Slobounov et al., 2005, p. 316).

Due to the rising size and complexity of data sets the use of predictive analyses using machine learning analysis became common in biomedical engineering studies (Poldrack et al., 2020). This results in the advantage of being able to use machine learning to examine large high-dimensional datasets for patterns in a brief period and derive quick and relevant conclusions. Regarding the classification models for machine learning, the goal is always to get generalizable models that can also predict other data sets that are not from the same data collection or environment (Scheinost et al., 2019, p. 37).

The recent measurement setup BioVRSea has been used for various research purposes, including the prediction of motion sickness or the evaluation of biomarkers for concussions (Recenti et al., 2021; Jacob et al., 2022). For this study, BioVRSea was used to explore brain activity, muscle activity, and center of mass to investigate changes in PC responses. With these biosignal recordings, features can be extracted and then used to classify the several segments of the experiment. In total, BioVRsea consists of six distinct phases with individual settings that have a unique influence on the subjects’ responses in terms of PC. Firstly, from baseline to PRE, we have a visual onset with the beginning of the sea simulation. From PRE to 25%, we have a motion onset synchronized with the sea simulation. This movement progressively increases at 50% and 75%. Finally, the induced postural control response is measured while abruptly switching off the movement while the sea simulation remains on. Therefore, feature extraction and analysis were performed for each phase with the aim to estimate and quantify these different physiological conditions and responses. Machine learning is of great impact as it helps to determine which biosignals are suitable to predict the different phases of the BioVRSea acquisition and which patterns can be found in the large amount of data collected.

## Materials and Methods

### Experimental protocol

BioVRSea is an innovative measurement setup for studying the physiology of PC. It mimics the sensation and gives the impression of being in a small boat on a rough sea. The goal of the experimental setup is to trigger a PC response and analyze the various biosignals during the acquisition.

**Figure 1:**
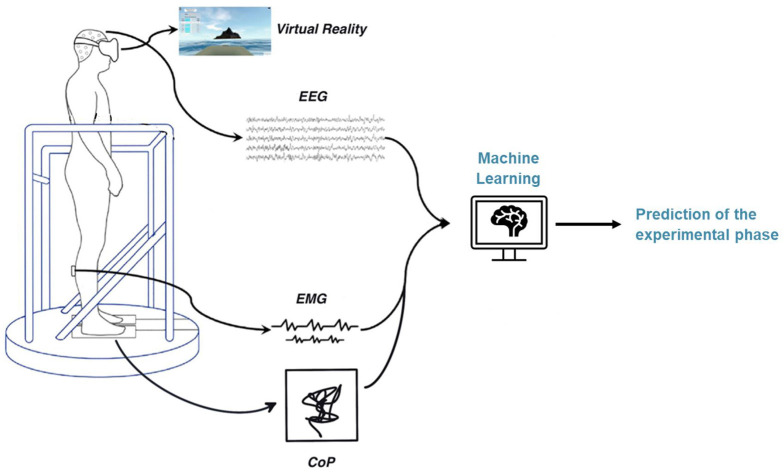
BioVRSea measurement setup.

The setup consists of a virtual reality (VR) environment and a moving platform synchronized with the simulated environment. Subjects stand on the platform and wear VR goggles and various measurement devices to record the different biosignals. The participants were prepared for measurement with the placement of a wet 64-electrode EEG cap, six wireless EMG sensors on the tibialis anterior, gastrocnemius lateral, and soleus muscles of each leg, and the heart sensor strapped around the chest. The EEG amplifier was connected to the cap and placed in a backpack with a tablet used for EEG signal acquisition. The backpack is worn by the participant during the experiment. Finally, the participant enters the VR environment by climbing on a platform and donning VR goggles. The platform is equipped with a force plate to measure the sway movements of the participants during the experiment. The schematic experimental setup can be seen in Figure 1.

**Figure 2:**
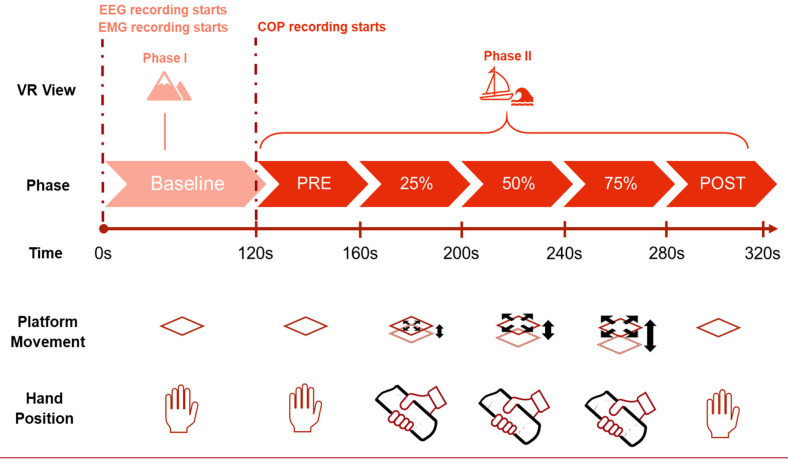
Different phases of the experiment.

For the experiment and the following classification, six phases are considered, as depicted in Figure 2. These segments will be the different classes to be predicted with the machine learning process. The first phase is the baseline segment, where the participant only sees a static mountain panorama through the VR goggles. During this time, the platform does not move, and the participant stands with their hands by their side while viewing the mountain view for a total of 2 min. For the signal processing part, only the last 60 s were used. After the Baseline phase, the VR scene changes to the sea environment and the subject sees him/herself on a small boat at sea. The second phase lasts 40 s and is called the PRE phase. During this phase, the platform does not move, and the participant remains still with their hands by their side. In the third phase, the participant holds on to the safety bars and the platform moves in a synchronized manner with the waves seen in the VR scene. During these 40 s, the platform moves at 25% of the maximum amplitude. In the fourth and fifth phases, the platform increases the intensity to 50% and 75%, respectively for 40 s each. In the last phase, the POST phase, the movement of the platform stops while the VR simulation continues. The participant takes their hands off the safety bar and stands quietly trying to maintain equilibrium watching the VR sea scene. After 40 s, this phase is over and so is the entire acquisition.

The different materials used for the study include the VR software (Sampling frequency: 90 Hz, Virtualis VR, Clapiers, France), the moving platform (Virtualis VR, Clapiers, France), the force platform (Virtualis VR, Clapiers, France), six wireless EMG sensors, attached to the soleus, tibialis anterioris, and gastrocnemius lateral on both the left and right legs (Sampling frequency: 1,600 Hz, Kiso ehf, Reykjavik, Iceland), and the 64-channel wet electrode cap (Sampling frequency: 4,096 Hz ANTNeuro, Hengelo, The Netherlands). The three integrated biosignals were recorded and synchronized using a software called MacroRecorder. This software enables to automatize all the consecutive computer tasks and to launch simultaneously the recording.

The data processing and analysis procedure are described in the following sections.

### Population

The study is based on 191 subjects (age: 34.1 ± 14.8), 79 male and 112 female subjects (ethics approval by the Icelandic Bioethics Commission—Number: VSN-20-101—May 2020).

Due to missing or inferior quality recordings, the CoP analysis was performed on only 172 subjects (age: 33.7 ± 14.7), 64 male and 108 female.

### EEG

Brainstorm (Tadel et al., 2011) and MATLAB 2022a (MATLAB, 2022; MathWorks, Inc., Natick, 158 Massachusetts, USA) with the Automagic Toolbox developed by Pedroni et al. (2019) was used for preprocessing and analysis of the EEG data. To ensure data quality and to avoid artifacts, we removed the five first and five last seconds for each phase of the experiment. Afterwards, the data were resampled to 1,024 Hz.

Automagic was used for automatic preprocessing for each data set, with a manual check at the end. The ICA MARA algorithm was used with a variance of 20%. A high-pass and low-pass filter were set to 1 Hz and 45 Hz, respectively. The data were notch filtered at 50 Hz. Finally, the bad electrodes were interpolated. According to the five anatomic lobes of the cortex consisting of frontal, parietal, occipital, and the two temporal lobes that are widely accepted in the scientific community (Woolsey et al., 2017; Casillo et al., 2020) we further used these lobes as regions of interest. The power spectral density (PSD) was computed for each phase and each electrode using Welch’s method, with the following frequency bands: delta (1–4 Hz), theta (4–8 Hz), alpha (8–13 Hz), beta (13–30 Hz), low gamma (30–45 Hz). The relative power of each region of the cerebral cortex was then calculated by taking the mean of the electrodes located in that region to obtain a total of five EEG-related features per region and per experimental phase. All this preprocessing was performed in a manner analogous to the study of Jacob et al. (2022).

### EMG

For the EMG features, preprocessing and feature extraction are required which were performed with MATLAB 2022a, using the built-in Signal Processing Toolbox. The muscle signal was analyzed for each segment. After the removal of the transition from one phase to another, each phase segment for 20 s. For further processing, we use a 4th order Butterworth bandpass, a cutoff frequency of 15 Hz and 500 Hz, and a sampling frequency of 1,600 Hz. Time and frequency metrics were extracted from this final signal. The full table with the used features can be found in Supplementary Figure S1.

### Centre of Pressure (CoP)

To calculate the anterior–posterior (AP) and medio-lateral (ML) displacement (in centimeters) of the CoP, the following formula was used:


$${\text{Displacement}}_{AP}=Y\times 0.5\times \text{StaticVR\_AP and}$$

$${\text{Displacement}}_{\text{ML}}=X\times 0.5\times \text{StaticVR\_ML}$$

The StaticVR_AP (respectively StaticVR_ML) is the percentage of the vertical size (respectively horizontal size) of the platform sensors, with a value comprised between −1 and 1. In addition, X represents the dimension of the force platform in the ML direction and Y represents the dimension of the force platform in the AP direction.

The processing of the CoP data was performed using MATLAB 2022a, and is the same as previously described in Jacob et al. (2022). During the experiment, the force platform records the movement of the CoP, a projection of the center of mass of the subject on the plane of the machine, also called a stabilogram. To filter the CoP data, we used a Savitsky-Golay filter with window size 7. Afterwards we used the stabilogram for feature extraction. The full list of the used features can be found in Supplementary Figure S1.

### Machine learning

For the classification analysis, the interactive Classification Learner App was used: it is included in the Statistics and Machine Learning Toolbox (2022) in MATLAB 2022a. The data can be imported as a spreadsheet, which contains the different predictor and response variables. We used a training dataset containing 80% of the data and a test dataset with the remaining 20%. As is known from machine learning theory and literature, it is crucial that the model has never seen the test data set until finally the test accuracy gets computed to avoid overfitting and biased results (Scheinost et al., 2019, p. 37; Poldrack et al., 2020).

For validation accuracy, we performed a 10-fold cross-validation and the validation error gets calculated by averaging over the 10 folds. Then, the model is trained using the entire training dataset (70% of the total data). Subsequently, we used the remaining 30% of the data to obtain test accuracy without bias.

As predictors, we used the features extracted from the different bio-signals (Table 1). To obtain information about which biosignal is more suitable for predicting the phase of the experiment, we trained and tested prediction models separately for the EEG, EMG, and CoP data. Finally, we also trained classification models using both EMG and CoP features together.

**Table 1:** Total number of features.

**Signal**	**Modalities**	**Features**	**Total**
**EEG**	5 bands × 5 brain regions = 25 modalities	1	25
**EMG**	3 muscles × 2 sides = 6 modalities	43	258
**CoP**	1 modality	35	35

EEG, electroencephalography; EMG, electromyography; CoP, center of pressure.

Different configurations have been used for the classification. The first one was the prediction of the six different phases. For the CoP data, the recording starts only after the BL, so the second configuration was the prediction of five phases (PRE, 25%, 50%, 75%, and POST). A third consideration was averaging the three movement phases (25%, 50%, 75%) in one unique phase, as the similarity of these phases made the classification more difficult. This leads to three (PRE, movement, POST) or four (BL, PRE, movement, POST) phases to classify. Finally, for the EEG signals, due to the low accuracy in the previous configurations, a binary classification was defined, to differentiate BL from the rest. The five phases that were not BL were averaged together in one single phase. This approach of merging was only possible because we had the same amount of data for each phase. This is also necessary for the classification to correctly assess and interpret the average overall accuracy (Poldrack et al., 2020).

The details of the classification configuration and results are listed in Table 2.

**Table 2:** Overview classification results and performance parameter.

	**Analyzed phases**											**Feature ranking**		
							**Validation**	**Test**	**AUC**					
**Bio-signal**	**BL**	**PRE**	**25**	**50**	**75**	**POST**	**accuracy [%]**	**accuracy [%]**	**validation**	**AUC test**	**Model**	**1.**	**2.**	**3.**
EEG	X	X					76.1	74.6	0.82	0.82	Subspace Discriminant	LG—occipital	A—parietal	A—frontal
	X	X	X	X	X	X	24.3	27.1	0.57	0.61	Linear SVM	A—frontal	A—parietal	A—temporal—right

EMG	X	X	X	X	X	X	43.6	39.4	0.73	0.74	Linear Discriminant	LTKEO—TA—Right	LTKEO—TA—Left	LDASDV—TA—Right
		X	X	X	X	X	42.8	42.3	0.72	0.71	Linear Discriminant	LTKEO—TA—Right	LTKEO—GL– Right	LDASDV—TA—Right
	X	X	X			X	63.0	57.6	0.83	0.8	Linear Discriminant	LTKEO—TA—Right	LDASDV—TA—Right	MFL– TA—Right

CoP		X	X	X	X	X	72.1	71.7	0.92	0.92	Linear SVM	RDIST—ML	SD– ML	Direction Entropy
		X	X			X	80.1	85.1	0.93	0.93	Subspace Discriminant	Direction Entropy	RDIST—ML	SD—ML

EMG and CoP		X	X	X	X	X	64.5	74.4	0.91	0.92	Medium Gaussian SVM	RDIST—ML	SD—ML	Direction Entropy
		X	X			X	83.1	83.8	0.96	0.95	Subspace Discriminant	Direction Entropy	RDIST—ML	SD—ML

AUC, area under curve; MFL, maximum fractal length; SVM, Support Vector Machine; LTKEO, logarithmic Teager-Kaiser energy operator; RDIST_ML, Squared Root Mean Distance of points on Medio-Lateral axis; TA, Tibialis Anterioris; LDASDV, logarithmic difference absolute standard deviation value.

For calculating the feature selection, the ANOVA—analysis of variance—method was used. ANOVA provides the opportunity to determine the most relevant feature for classification. As a statistical test, it is suitable to compare the differences between two or more means of the samples based on mean and variance. The ones with the lowest values show that they are independent of the target variable. The ones with a high value show a high relevance for the classification model. ANOVA is suitable for non-stationary data such as EEG for example and is used in various other studies in the field of biomedical engineering and neuroscience (Chowdhury et al., 2017, p. 537; Majid Mehmood et al., 2017, p. 674; Harpale and Bairagi, 2021, p. 14,797).

Afterwards, the effect size has been computed, calculating η^2^ for a confidence level of 95%.

## Results

Table 2 sums up the classification results. The first column shows the biosignal used for the prediction. The second column shows the phases considered in the analyses. The “X” overlapping several cells, as on the first row of EEG, considers the merged phases. The next two columns represent the validation and test accuracy, followed by the area under curve (AUC) for the validation and test. The seventh column shows which model has been used to obtain those results, and the last three columns show the top three features for the classification.

### Phases classification

**Figure 3:**
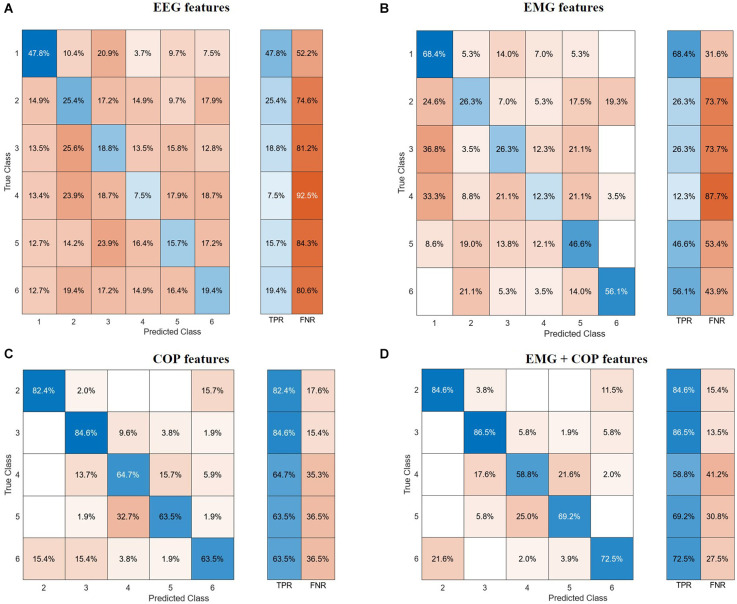
Confusion matrices. **(A)** EEG features. **(B)** EMG features. **(C)** CoP features. **(D)** EMG and CoP features. EEG, electroencephalography; EMG, electromyography; CoP, center of pressure.

The confusion matrices in Figure 3 visualize and summarize the performances of the models detailed in Table 2, for each biosignal: EEG, EMG, COP, and EMG+COP. The table layout shows two dimensions, the true class in each row and the predicted class in each column, and gives insights into the occurrence frequency in the corresponding field. Furthermore, the True Positive Rates (TPR) and the False Negative Rates (FNR) are shown next to that.

With the EEG features, it is not possible to classify all the different phases of the experiment. But with a test accuracy of 74.6% it is possible to classify if the data is from Baseline or not. Regarding the use of EMG features with our test data, we could predict with an accuracy of 68.4% of the Baseline. As can be seen in Figure 3B, it is difficult for the models to classify the phases in between, resulting in high false negative rates for these phases.

Using the CoP features we can classify five phases with a test accuracy of 71.7%. Striking is the True Positive Rate of 82.4% for predicting the PRE-Phase and 84.6% for the 25% phase as can be seen in Figure 3C. Due to the False Negative Rate of 36.5% for the Post phase, it becomes clear that the model has more issues to predict the POST phase by only using the CoP data. Most of the mistakes in predicting the phase result in the fact that in 15.7% of the cases the predicted class is the PRE phase instead of the POST phase. Respectively this correlation can also be seen if the model tries to predict the PRE-Phase.

By using both EMG and CoP features we get the best result for predicting the five different phases. For the trained model we achieved a test accuracy of 74.4% and an AUC value of 0.92. The single True Positive and the respective False Negative Rates can be seen in Figure 3D.

The use of EEG, EMG, and CoP features jointly did not bring an increase in the overall accuracy for predicting five phases of the experiment compared of the use of EMG and CoP features together.

### Features ranking

**Figure 4:**
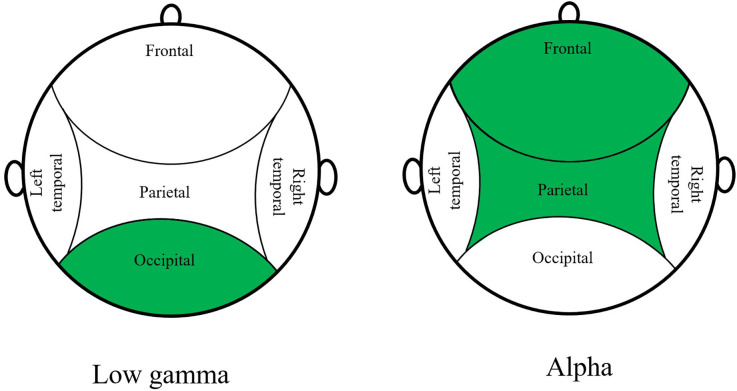
Top 3 EEG features to classify BL from the experiment.

The three most relevant EEG features to classify the BL from the experiment are the power spectral density values for the low-gamma phase in the occipital region, the alpha phase in the frontal region, and in the parietal region, represented in Figure 4.

If only the EMG data is used, the most useful feature is the logarithmic Teager-Kaiser energy operator (LTKEO) of the Tibialis Anterioris Right, followed by LTKEO of the Tibialis Anterioris Left and the logarithmic difference absolute standard deviation value (LDASDV) of the Tibialis Anterioris Right.

**Figure 5:**
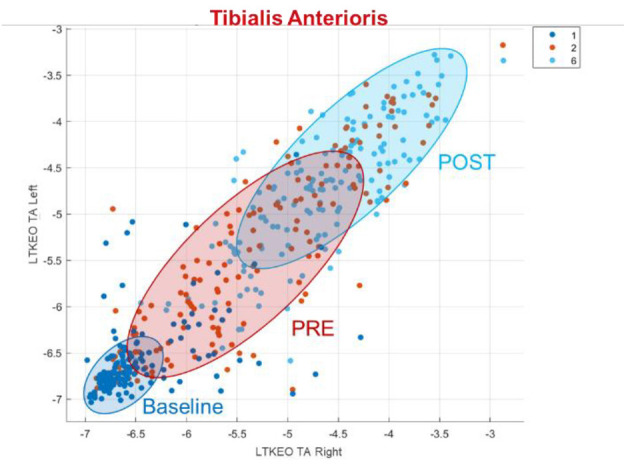
LTKEO feature for tibialis anterioris. LTKEO, Logarithmic Teager-Kaiser energy operator.

The LTKEO feature plotted for the Tibialis Anterior and for Baseline, PRE and POST can be seen in Figure 5. An increase is recognizable in the LTKEO feature with the ongoing experiment. Strikingly is also the concentrated clustering for Baseline and more widely distributed data dots for the PRE and POST phases.

Regarding CoP, the three most relevant features for the classification algorithms are the squared root mean distance of the ML direction (RDIST_ML) as well as the standard deviation of points on the ML axis (SD ML), and the direction entropy. In general, it can be noticed that the ML features are more relevant to predict the phases than the AP features.

**Figure 6:**
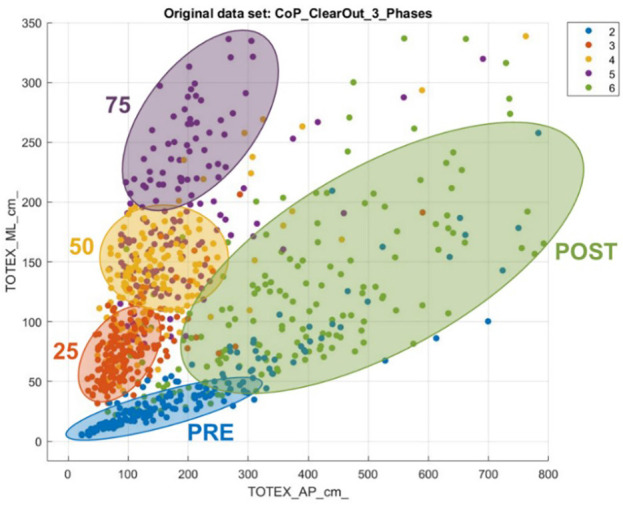
Total CoP movement in anterior-posterior and medio-lateral direction.

The different phases of the experiment can be seen in Figure 6, plotting the total CoP movement in AP Direction and ML Direction. The PRE-Phase strikes out with the lowest values for the total movement regarding both directions. Furthermore, the data dots for the PRE-Phase are limited and concentrated in a small area. With the experiment’s further progress, a stepwise increase is noticeable in the Medio-Lateral direction and a slight increase in the Anterior-Posterior direction. The finishing POST-phase characterizes itself with increased values in the Anterior-Posterior direction and a widely distributed area.

Table 3 describes the effect size for the top three features of each biosignal. EEG features have an effect size of around 0.05, highlighting a medium effect. However, EMG features have an effect size of 0.4, and COP features have an effect size of 0.5, showing a high effect associated with those features.

**Table 3:** Effect size description.

**Effect size**			
**EEG**	**LG occipital**	**A parietal**	**A frontal**
**η^2^**	0.04	0.05	0.07
**EMG**	**LTKEO–TA–Right**	**LTKEO–TA–Left**	**LDASDV–TA–Right**
**η^2^**	0.41	0.39	0.41
**COP**	**RDIST–ML**	**SD–ML**	**Direction Entropy**
**η^2^**	0.53	0.53	0.50

## Discussion

Postural control is a sensorimotor mechanism that can reveal neurophysiological disorder. The main goal of using the classification is to gain more insight into the dynamic changes in posture control and the different phases of the experiment. This is the reason behind the classification of the different phases of BioVRSea.

### EEG features

With the EEG feature solely, we can only classify Baseline and not Baseline with an accuracy of 74.6%. This shows that there is a change in brain activity and a difference between those two stages is recognizable for the model. This difference can be deduced from the changing VR scene that also triggers the occipital region of the brain. This conclusion may fit together with the PSD value for the low gamma phase in the occipital region that is the most important feature for binary classification.

The attempt to classify all six phases was not successful. Considering the experimental setup this also seems reasonable as the phases of the moving platform (25%, 50%, and 75%) will trigger the same regions (such as the frontal and parietal regions) as they are needed for coordination and motion control (Faw, 2003; Fogassi and Luppino, 2005; Aubonnet et al., 2022). This makes it challenging for a classification model to predict a class. Another possible reason is the individual adaptive PC strategy to the sensory input regarding the neural activity (Ivanenko and Gurfinkel, 2018).

Other causes for the difficulties in predicting all the phases could be that in the study we took the average PSD value for the entire phase. An improvement could be to take epochs, so that averaging the different spikes on a per-wave basis could give a more appropriate feature extraction method. Including features derived from a dynamic analysis (not static as reported here) may also improve performance.

### EMG interpretation

Regarding the EMG features, the high FNR for the phases 25%, 50%, and 75% is expectable as they differ only in their intensity of the sensory input. As the Baseline is the most different phase compared to the others (no sea simulation, no platform movements), it makes sense that it is also for the model one of the easiest phases to predict.

The logarithmic Teager-Kaiser energy operator (LTKEO) of the Tibialis Anterioris is the most important feature for the classification with solely EMG features. It leads to a good classification result, especially between Baseline and the other phases. Li et al. (2007) discovered an improved detection of EMG onset using TKEO, especially at a low signal-to-noise ratio. Other studies confirmed this, reasoning that the calculated energy is derived from the instantaneous amplitude and frequency of the signal (Solnik et al., 2010; Liu et al., 2015). Laksono et al. (2021) also performed EMG classification using the Matlab classification learning application and found an important role of TKEO for EMG classification and high accuracy.

The overlapping clusters for the PRE and the POST phase in Figure 5 show that the LTKEO values are on average higher in the POST phase but it also shows that this feature on its own is not sufficient to distinguish these two Phases without mistakes. The reason the LTKEO values are so low in the Baseline could be that it is the easiest phase to maintain PC as the VR environment with the static mountain view combined with the platform not moving is not a challenging task for subjects without PC disorders. In that phase, the muscles are relaxed all the time, and the subjects rarely contract their muscles to keep balance. This leads to low instantaneous energy changes and therefore a low LTKEO value.

A hypothesis for the widely distributed dots in PRE and POST visible in Figure 5 is that some of the people are using more balancing movements in those two segments, compared to others that just need less muscle activity to maintain PC. This quantitively underlines the fact that people are using individual adaptive PC strategies as identified in the literature (van Emmerik and van Wegen, 2002).

### CoP interpretation

A similar interpretation can be reached with the CoP data. Figure 6 shows how severe and individual the impact is on the subjects’ PC. The increase in deviation from the neutral position indicates the substantial influence that the experiment had on most of the subjects. While observing the scattered plot, the post phase is more widely distributed, because the severeness of the impact on the subjects is quite different. This result also must be seen with the background that anatomical prerequisites also affect the adaptive strategies and are why some people show a higher deviation from the neutral position than others (Hunter and Hoffman, 2001).

The fact that more ML features are relevant for the classification leads to the conclusion that, for these features, the experiment results in patterns that differ among each phase, and help the classification model to predict. A clinical hypothesis for it could be that sways in the AP direction are more individual and natural than sways in the ML direction that are triggered by our acquisition (Hunter and Hoffman, 2001). In addition to that, the VR environment is mainly an AP visual oscillation that is active in all the phases besides Baseline. This could make it more difficult to predict the phase as th differences between the phases are less significant. This result also matches with other findings from previous studies (Lott et al., 2003; Donker et al., 2007; Luo et al., 2018).

### EMG and CoP interpretation

By combining EMG and CoP measurements we obtain good classification results, with 73.3% of accuracy for five phases. The reason could be that with the EMG data it is difficult for the model to distinguish the phases of 25%, 50%, and 75%. For these phases, the model can use the CoP data to classify them. This leads to high overall accuracy.

Comparing the different biosignal features it becomes clear that EEG is not able to predict all the phases for the BioVRSea experiment. With EMG as well, the results are not sufficient. With CoP superior results can be achieved but really satisfying results for the classification of the distinct phases can only be achieved by using EMG and CoP features jointly. This leads to the result that those biosignals are needed to describe the dynamic behavior BioVRSea has on the subjects and to quantitatively assess the adaptive PC strategies.

### Limitations

The first limitation to mention could be the amount of data. This is relatively large for a typical biomedical study with neurophysiological examinations, but the results, especially the learned classification models, could be even more reliable if the amount of data were larger. This is especially critical when subgroups are studied, as the number of subjects is then greatly reduced. In addition, the diversity of the subjects investigated should be increased in further studies. Also, relative to the average population, more women than men participated in the study.

Another limitation was the subjects’ behavior during the experiment. Even though we instructed them to stand still and look in front of them, some participants performed spontaneous movements that were not in reaction to the experiment. This could lead to artifacts or noise in the data collection. Also, it cannot be fully ensured that subjects remain fully focused on the experiment and do not pursue other conscious thoughts.

## Conclusion

This work aimed to study the neurophysiological dynamics of PC, and how neurophysiological measures could help to quantify and predict PC adaptation, from a consequent healthy cohort. We demonstrated that for our paradigm, EEG could differentiate baseline from acquisition phases. Moreover, the combination of EMG and CoP parameters presented satisfactory results to characterize the five phases of the experiment involving the sea simulation. From those results, the best features of each signal were identified to classify the phases: alpha frontal and parietal PSD, lowgamma occipital PSD for the EEG, LTKEO for tibialis anterioris for the EMG, and RDIST and SD ML for the CoP.

This work is a first step towards the definition of a global healthy pattern, and will lead in the future to the development of tools to understand and quantify PC-related pathological conditions.

## Data Availability Statement

The raw data supporting the conclusions of this article will be made available by the authors, without undue reservation.

## Ethics Statement

The studies involving human participants were reviewed and approved by Icelandic Bioethics Commission Number: VSN-20-101. The patients/participants provided their written informed consent to participate in this study.

## Author Contributions

SS and RA wrote the manuscript. SS performed the machine learning pipeline and EMG and COP analysis. RA performed the EEG analysis. DJ helped with EMG and COP analysis and reviewed the manuscript. MR and MH helped with the machine learning pipeline and reviewed the manuscript. HP helped with the results discussion and reviewed the manuscript. PG supervised the work and reviewed the manuscript. All authors contributed to the article and approved the submitted version.

## Abbreviations

A: alpha
AP: antero-posterior
AUC: area under curve
B: beta
CoP: center of pressure
FNR: false negative rate
LDASDV: logarithmic difference absolute standard deviation value
LG: low gamma
LTKEO: logarithmic Teager-Kaiser energy operator
ML: medio-lateral
PC: postural control
RDIST_ML: Squared Root Mean Distance of points on ML axis
TPR: true positive rate.
