# Supplementary material for: Predicting postural control adaptation measuring EEG, EMG, and center of pressure changes: BioVRSea paradigm
Source: Front Hum Neurosci. 2022 Dec 15;16:1038976. doi: 10.3389/fnhum.2022.1038976 (PMC9797538; doi:10.3389/fnhum.2022.1038976)
Supplement: Supplementary file 1 [file Data_Sheet_1.PDF]

Supplementary Material

1 Supplementary Figures and Tables

1.1 Supplementary Figures

Feature	Formula	
Average Amplitude Change (AAC)	$AAC = \frac{1}{N} \cdot \sum_{i=1}^{N-1} \lvert x_{i+1} - x_i \rvert$	
Average Energy (AE)	$AE = \frac{1}{N} \cdot \sum_{i=1}^{N} (x_i)^2$	
Absolute Value of the Summation of the Exponential Root (ASM)	$ASM = \frac{1}{N} \cdot \sum_{i=1}^{N} \lvert (x_i)^p \rvert$	$p = \begin{cases} 0.5\ if\ 0.25N \le i \le 0.75N \\ 0.75\ otherwise \end{cases}$
Absolute Value of the Summation of Square Root (ASS)	$ASS = \sum_{i=1}^{N} \sqrt{\lvert x_i \rvert}$	
Coefficient of Variation (CV)	$CV = \frac{SD}{\bar{x}}$	
Difference Absolute Mean Value (DAMV)	$DAMV = \frac{\sum_{i=1}^{N-1} \lvert x_{i+1} - x_i \rvert}{N-1}$	
Difference Absolute Standard Deviation Value (DASDV)	$DASDV = \sqrt{\frac{\sum_{i=1}^{N-1} (x_{i+1} - x_i)^2}{N-1}}$	
Difference Variance Value (DVARV)	$DVARV = \frac{1}{N-2} \cdot \sum_{i=1}^{N-1} (x_{i+1} - x_i)^2$	
Enhanced mean absolute value (EMAV)	$EMAV = \frac{1}{N} \cdot \sum_{i=1}^{N} \lvert (x_i)^p \rvert$	$p = \begin{cases} 0.75\ if\ 0.2N \le i \le 0.8N \\ 0.5\ otherwise \end{cases}$
Enhanced wavelength (EWL)	$EWL = \sum_{i=2}^{N} \lvert (x_i - x_{i-1})^p \rvert$	$p = \begin{cases} 0.75\ if\ 0.2N \le i \le 0.8N \\ 0.5\ otherwise \end{cases}$
New Zero Crossing (FZC)	$FZC = \sum_{i=1}^{N-1} w_i$	$T = \frac{2}{5} \cdot \sum_{i=1}^{10} x_i$

**Feature**	**Formula**	
		$w_i = \begin{Bmatrix} 1\ if\ x_i > T\ \&\ x_{i+1} < T \\ 1\ if\ x_i < T\ \&\ x_{i+1} > T \\ 0\ otherwise \end{Bmatrix}$
Kurtosis (KURT)	$KURT = \frac{\bar{x}^4}{SD^4}$	
Integrated EMG (IEMG)	$IEMG = \sum_{i=1}^{N} \lvert x_i \rvert$	
Interquartile Range (IQR)	$IQR = Q_3 - Q_1$	
Log CV (LCV)	$LCOV = \log(COV)$	
Log detector (LD)	$LD = exp\left(\frac{1}{N} \cdot \sum_{i=1}^{N} \log\lvert x_i \rvert\right)$	
Log DAMV (LDAMV)	$LDAMV = \log(DAMV)$	
Log DASDV (LDASDV)	$LDASDV = \log(DASDV)$	
Log Teager Kaiser Energy Operator (LTKEO)	$LTKEO = \log\left(\sum_{i=2}^{N-1} (x_i)^2 - x_{i-1} \cdot x_{i+1}\right)$	
Mean Absolute Deviation (MAD)	$MAD = \frac{1}{N} \cdot \sum_{i=1}^{N} \lvert x_i - \bar{x} \rvert$	
Mean Absolute Value (MAV)	$MAV = \frac{1}{N} \cdot \sum_{i=1}^{N} \lvert x_i \rvert$	
Maximum Fractal Length (MFL)	$MFL = \log\left(\sqrt{\sum_{i=1}^{N-1} (x_{i+1} - x_i)^2}\right)$	
Modified Mean Absolute Value (MMAV)	$MMAV = \frac{1}{N} \cdot \sum_{i=1}^{N} w_i \cdot \lvert x_i \rvert$	$w_i = \begin{Bmatrix} 1\ if\ 0.25N \leq i \leq 0.75N \\ 0.5\ otherwise \end{Bmatrix}$

Feature	Formula	
Modified Mean Absolute Value 2 (MMAV2)	$MMAV2 = \frac{1}{N} \cdot \sum_{i=1}^{N} w_i \cdot \lvert x_i \rvert$	$w_i = \begin{Bmatrix} 1\ if\ 0.25N \le i \le 0.75N \\ \frac{4i}{N}\ if\ i < 0.25N \\ \frac{4(i-N)}{N}\ otherwise \end{Bmatrix}$
Mean Value of the Square Root (MSR)	$MSR = \frac{1}{N} \cdot \sum_{i=1}^{N} \sqrt{x_i}$	
Root Mean Square (RMS)	$RMS = \sqrt{\frac{1}{N} \cdot \sum_{i=1}^{N} (x_i)^2}$	
Standard Deviation (SD)	$SD = \sqrt{\frac{1}{N-1} \cdot \sum_{i=1}^{N} (x_i - \bar{x})^2}$	
Skewness (SKEW)	$SKEW = \frac{\sum_{i=1}^{N} (x_i - \bar{x})^3}{(N-1) \cdot SD^3}$	
Single Square Integral (SSI)	$SSI = \sum_{i=1}^{N} (x_i)^2$	
Absolute Value of Temporal Moment (TM)	$TM = \frac{1}{N} \cdot \sum_{i=1}^{N} \lvert (x_i)^p \rvert$	$p \in N$ $commonly\ p = 3$
Variance (VAR)	$VAR = \frac{1}{N-1} \cdot \sum_{i=1}^{N} (x_i - \bar{x})^2$	
Variance of EMG (VARE)	$VARE = \frac{1}{N-1} \cdot \sum_{i=1}^{N} (x_i)^2$	
Variance Order (VO)	$VO = \sqrt{\frac{1}{N} \cdot \sum_{i=1}^{N} (x_i)^p}$	$p \in N$ $commonly\ p = 2$
Waveform Length (WL)	$WL = \sum_{i=2}^{N} \lvert x_i - x_{i-1} \rvert$	

Feature	Formula
Power (p_i)	$p_i = \frac{1}{M} \cdot (X_i)^2$
Mean Frequency (F_{MN})	$F_{MN} = \frac{\sum_{i=1}^{M} f_i \cdot p_i}{\sum_{i=1}^{M} p_i}$
Median Frequency (F_{MD})	$F_{MD} = \frac{1}{2} \cdot \sum_{i=1}^{M} p_i$
Total Power (P_T)	$P_T = \sum_{i=1}^{M} p_i$
Mean Power (P_{MN})	$P_{MN} = \frac{1}{M} \cdot \sum_{i=1}^{M} p_i$
Peak Power (P_{max})	$P_{max} = \max(p_i)$
Peak Frequency (F_{max})	$F_{max} = \max(f_i)$
Kurtosis Frequency (F_{kurt})	$F_{kurt} = \frac{(F_{MN})^4}{(F_{SD})^4}$
Skewness Frequency (F_{skew})	$F_{skew} = \frac{\sum_{i=1}^{M} (f_i - F_{MN})^3}{(M-1) \cdot F_{SD}^{\ 3}}$

Supplementary Figure S1. List of all EMG features

1.2 Supplementary Tables

Supplementary Table S1. List of all CoP features

Feature Name	Description	Units
TOTEX	Summation over the elementary movement between consecutive samples on the support plane.	cm
TOTEX_ML	Summation over the elementary movement between consecutive samples on the ML axis.	cm
TOTEX_AP	Summation over the elementary movement between consecutive samples on the AP axis.	cm
RD	Summation of the square root distance between a point at time t and the plane origin.	cm
MDIST_ML	Mean of the distance of points with respect to the origin along the ML axis.	cm
MDIST_AP	Mean of the distance of points with respect to the origin along the AP axis.	cm
MVELO	Mean Velocity of points on the support plane, computed as TOTEX / Total Time.	cm/s
MVELO_ML	Mean Velocity of points on the ML axis, computed as TOTEX_ML / Total Time.	cm/s
MVELO_AP	Mean Velocity of points on the AP axis, computed as TOTEX_AP / Total Time.	cm/s

RDIST	Squared Root Mean Distance of points on the support plane with respect to the origin.	cm
RDIST_ML	Squared Root Mean Distance of points on ML axis with respect to the origin.	cm
RDIST_AP	Squared Root Mean Distance of points on AP axis with respect to the origin.	cm
ML SampEn	Sample Entropy computed on ML axis	nats
AP SampEn	Sample Entropy computed on AP axis	nats
ML CI	Summation of SampEn computed with increasing time scale on ML axis.	nats
AP CI	Summation of SampEn computed with increasing time scale on AP axis.	nats
Ellipse Area	Area of the 95% confidence ellipse.	cm^2
EllipseAngle	Angle between main axis of the 95% confidence ellipse and ML axis.	deg
EllipseMainAxisLength	Length of the Main Axis of the 95% confidence ellipse.	cm
EllipseMinorAxisLength	Length of the Minor Axis of the 95% confidence ellipse.	cm
SD AP	Standard deviation of points on the AP axis	cm
SD ML	Standard deviation of points on the ML axis	cm
SD Magnitude	Standard deviation of the distance of points with respect to the origin	cm
SD Direction	Standard deviation of the angle difference between successive points with respect to the ML axis	deg
Magnitude Entropy	SampleEntropy of the distance of points with respect to the origin	nats
Direction Entropy	SampleEntropy of the angle difference between successive points with respect to the ML axis	nats
Multivariate CI	Summation of SampEn computed with increasing time scale on the multivariate ML-AP series.	nats
AnteroMagnitude	Magnitude of the furthest point in the anterior portion of the plane.	cm
AnteroAngle	Angle of the furthest point in the anterior portion of the plane.	deg
PosteroMagnitude	Magnitude of the furthest point in the posterior portion of the plane.	cm
PosteroAngle	Angle of the furthest point in the posterior portion of the plane.	deg
LeftMagnitude	Magnitude of the furthest point in the left portion of the plane.	cm
LeftAngle	Angle of the furthest point in the left portion of the plane.	deg
RightMagnitude	Magnitude of the furthest point in the right portion of the plane.	cm
RightAngle	Angle of the furthest point in the right portion of the plane.	deg
